# Identifying biomarkers of the gut bacteria, bacteriophages and serum metabolites associated with three weaning periods in piglets

**DOI:** 10.1186/s12917-022-03203-w

**Published:** 2022-03-17

**Authors:** Xinwei Xiong, Xianxian Liu, Zhangfeng Wang, Qiao Xu, Jiguo Xu, Yousheng Rao

**Affiliations:** 1 Institute of Biological Technology, Nanchang Normal University, Nanchang, Jiangxi 330032 People’s Republic of China; 2 Key Laboratory of Women’s Reproductive Health of Jiangxi, Jiangxi Provincial Maternal and Child Health Hospital, Nanchang, Jiangxi 330006 People’s Republic of China

**Keywords:** Piglet, Metagenomic sequencing, Gut bacteria, Bacteriophages, Serum metabolomics, Weaning

## Abstract

**Background:**

The establishment of the piglet gut microbiome has a prolonged influence on host health, as it sets the stage for establishment of the adult swine microbiome. Substantial changes in host metabolism and immunity around the time of weaning may be accompanied by alterations in the gut microbiome. In this study, we systematically evaluated differences in the gut microbiome and host metabolites among three weaning periods using shotgun metagenomic sequencing and untargeted metabolomic profiling in piglets.

**Results:**

We identified that *P. copri* was the most significantly different species among three weaning periods, and was the key bacterial species for mitigating piglet adaptation during the weaning transition, while Bacillus_phage_BCD7, the only differential bacteriophages, was significantly and positively correlated with *P. copri* enriched in day 28 group. Additionally, *P. copri* and Bacillus_phage_BCD7 was significantly correlated with the shifts of functional capacities of the gut microbiome and several CAZymes in day 28 group. Furthermore, the altered metabolites we observed were enriched in pathways matched to the functional capacity of the gut microbiome e.g., aminoacyl-tRNA biosynthesis.

**Conclusion:**

The results from this study indicate that the bacteria-phage interactions and host-microbial interactions during the weaning transition impact host metabolism, leading to beneficial host changes among three weaning periods.

**Supplementary Information:**

The online version contains supplementary material available at 10.1186/s12917-022-03203-w.

## Background

The gut microbiome is closely related to host health and plays a key role in digestion, nutrient absorption, physiological functions, and immune regulation [1, 2]. The bacterial composition and diversity of the gut have been shown to be affected by numerous factors, including host diet, medications, presence of pets, socioeconomic status, residence environment, and chance acquisition of lineages [3–11]. Moreover, the establishment of the infant gut microbiome early in life sets the stage for the characteristics of the adult microbiome and therefore has a prolonged influence on host health [12, 13]. Early-weaning-induced stress causes diarrhea, thereby increasing mortality and reducing growth performance in piglets [14]. Therefore, research that aims to better understand the composition and succession of gut microbiota in piglets is necessary for pig health.

The gut virome, also known as the phageome, is defined as the portion of the intestinal microbiome representing viruses that target either bacteria, fungi, or archaea, and most of them are bacteria viruses (bacteriophages or phages) [15]. Bacteriophages are the most abundant biological entities on earth and have a major impact on microbial communities [16, 17]. In addition, bacteriophages can increase bacterial growth and fitness by providing bacteria with genes for touching upon polysaccharide, toxin, carbohydrate metabolism and antibiotic resistance [18, 19]. Kim and Bae (2018) discovered bacteriophages can destroy the host cells and modify host phenotypes through lysogenic conversion besides alternate the bacterial communities by infection [20]. These studies suggested phages are to play important roles in shaping the bacterial community structure of the gut and bacteria-phage interactions are central to the bacterial physiology or metabolism.

Weaning is a special and important event for piglets and presents a challenge to piglet gut physiology [21]. To date, few studies have focused on the development of the gut microbiome during the suckling and weaning period. A study focused on gradual changes in the gut microbiota of weaned miniature piglets was recently done [22], but it did not study the gut microbiota of piglets during the suckling period. Another similar study was only used 16S rRNA gene sequencing [23]. Also, Frese et al. found that the gut microbiome and functional capacities are dramatically shaped in before and after weaning using 16S rRNA gene sequencing and metagenomic sequencing [24]. However, relatively little is known about the bacteria-phage interactions and host-microbial interactions during the weaning transition. In this study, we collected the feces and serum samples from pigs at three different ages, 14, 21, and 28 days during weaning periods. Then, shotgun metagenomic sequencing was performed on fecal samples and untargeted metabolomic profiles of host serum samples were measured to comprehensively characterize porcine fecal bacterial and virus composition, functional capacity, and serum metabolites during weaning periods. Using this approach, we identified bacterial and virus taxa and KEGG pathways in the gut microbiome that were significantly influenced by weaning periods. Furthermore, we detected a subset of serum metabolites that had altered abundance during weaning periods.

## Results

### Gut bacteria differences among three weaning periods

Five Large White piglets (two male and three female) across three age strata were studied. Fecal sampling continued for all five piglets at 14 days (day 14 group), 21 days (day 21 group, the day of weaning), and 28 days (day 28 group) of age. The sequence assembly analysis of 15 samples produced a total of 1,566,160 contigs with an average length of 2,090 bp and an average N50 length of 4,599 bp (Supplementary Table S1). The phylogenetic composition of the fecal bacteria were determined by blasting against the National Center for Biotechnology Information (NCBI) non-redundant (NR) database.

**Fig. 1:**
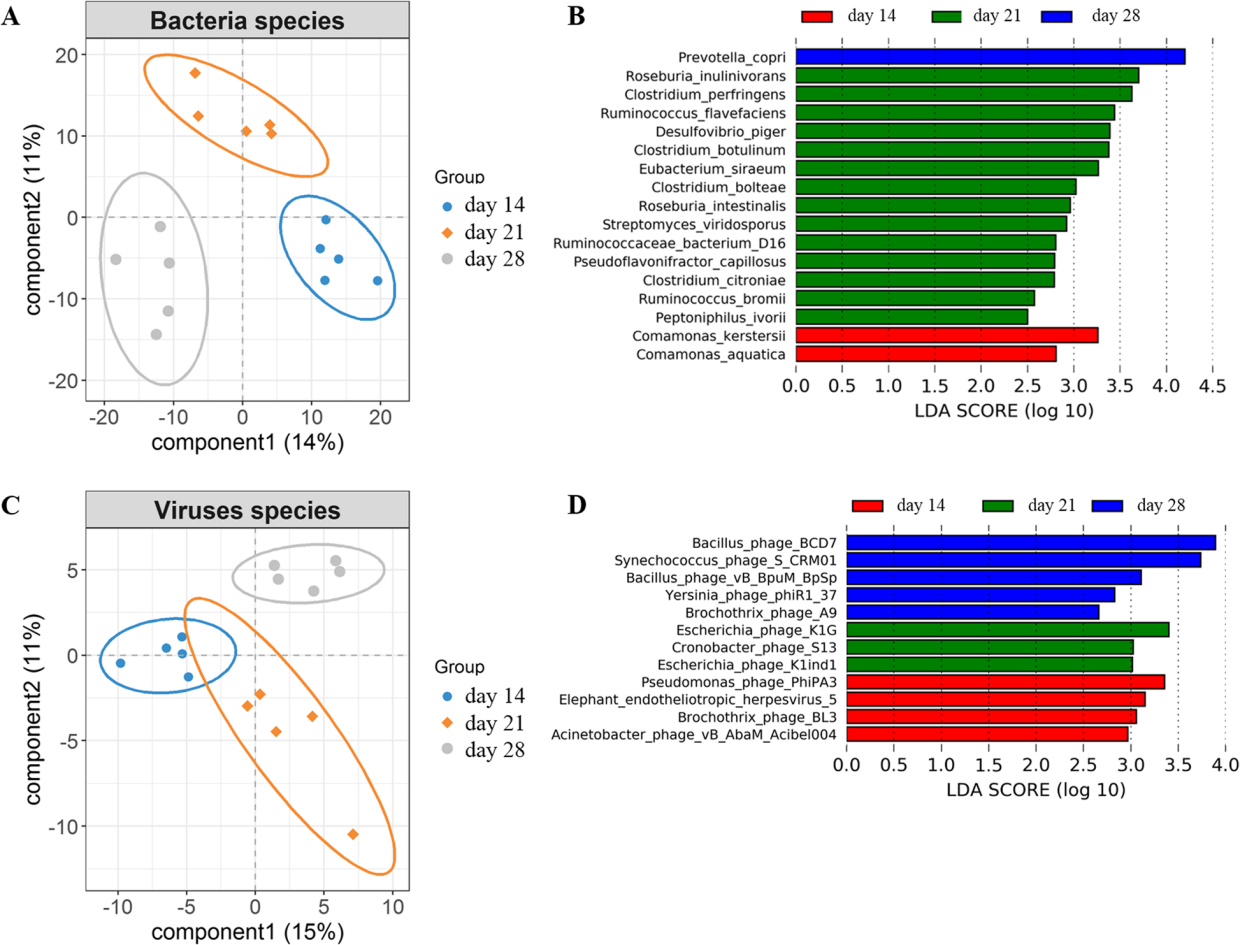
Gut microbiome characteristics among three weaning periods. **A** PLS-DA plot of fecal bacterial species, which indicates the significant differentiation of gut bacterial species among the three weaning periods. **B** The differential gut bacterial species. **C** PLS-DA plot of fecal virus species, which indicates the significant differentiation of gut virus species among the three weaning periods. **D** The differential gut bacteriophages

**Fig. 2:**
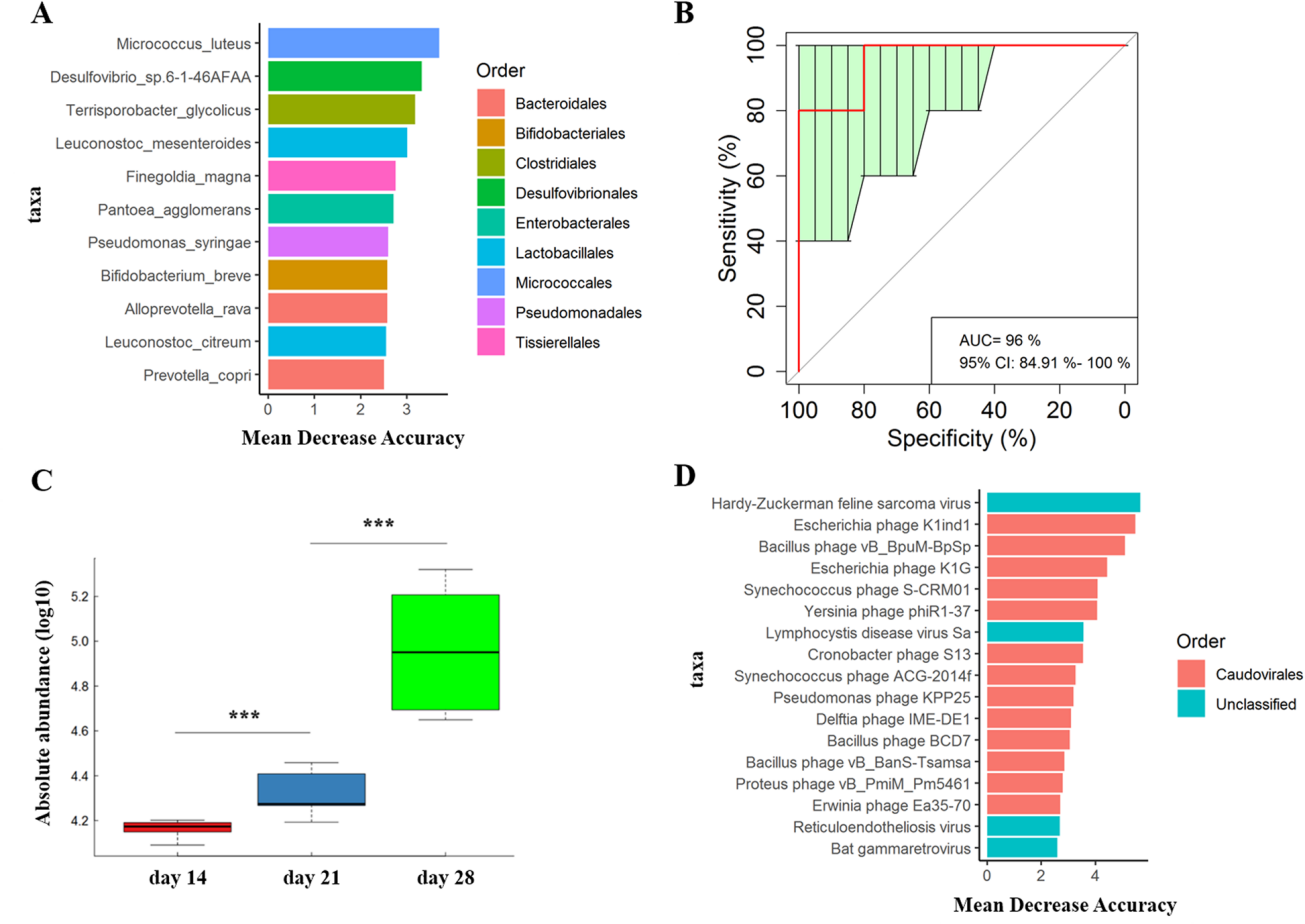
The changes in gut bacterial species and bacteriophages based on metagenomic sequencing results among the three weaning periods. **A** Random forest analysis to determine our ability to discriminate samples from different weaning periods based on gut bacterial species. **B** Receiver operating curve (ROC) of *P. copri*. The AUC was 96% with the 95% CI of 84.91–100%. **C** The absolute abundances of *P. copri*. **D** Random forest analysis to determine our ability to discriminate samples from different weaning periods based on gut virus species

We identified 55 phylum and 435 genus in all 15 samples. *Prevotella* and *Bacteroides* were the two most abundant genera. At the species level, a total of 746 bacterial species were detected in all 15 samples. *Bacteroides fragilis* was the most abundant bacterium in the tested samples. Initially, PLS-DA showed that bacterial species were an obvious shift (Fig. 1A). Furthermore, a total of 17 bacterial species were identified to be significantly different among the three weaning periods (Fig. 1B), including two species significantly enriched in the day 14 group and 14 species significantly enriched in the day 21 group. For example, *Comamonas kerstersii* and *Comamonas aquatica* and *Roseburia inulinivorans*, *Clostridium perfringens,* and *Ruminococcus flavefaciens* were enriched in the day 14 and day 21 groups, respectively. Specifically, *P. copri* was the only significantly different species enriched in the day 28 group. Subsequently, a random forest analysis was performed to examine our ability to discriminate samples from different weaning periods based on fecal microbiota metagenomic sequencing at species level (Fig. 2A). The results also showed that *P. copri* was distinct species among three weaning periods. And *P. copri* could distinguish three weaning periods with robust and high diagnostic accuracy of the area under the curve (AUC) 96% (Fig. 2B). Furthermore, as compared to those from day 21 group, the *P. copri* individuals from day 28 group had a significantly higher abundance (*P* < 0.001, FDR) while those from day 14 group had a significantly lower abundance (*P* < 0.001, FDR) (Fig. 2C).

### Bacteriophage differences among three weaning periods

The metagenomic sequencing data was also mapped to the viral genomes in NCBI NR database. We identified one phylum, 60 genus and 175 species in all 15 samples. An obvious shift in the gut virus species was observed among three weaning periods (Fig. 1C). Moreover, we identified a total of 12 discriminative bacteriophages among three weaning periods (Fig. 1D), including five bacteriophages that were significantly enriched in the day 28 group, such as Bacillus_phage_BCD7, Synechococcus_phage_S_CRM01 and Bacillus_phage_vB_BpuM_BpSp. We also identified four bacteriophages significantly enriched in the day 14 group and three bacteriophages significantly enriched in the day 21 group, including Pseudomonas_phage_PhiPA3, Elephant_endotheliotropic_herpesvirus_5, Brochothrix_phage_BL3, and Acinetobacter_phage_vB_AbaM_Acibel004 and Escherichia_phage_K1G, Cronobacter_phage_S13, and Escherichia_phage_K1ind1, respectively. A random forest analysis was then conducted to examine our ability to discriminate among the weaning periods based on fecal microbiota metagenomic sequencing of bacteriophages (Fig. 2D). As was found in LEfSe analysis, Bacillus_phage_BCD7, Escherichia_phage_K1ind1, Escherichia_phage_K1G, Synechococcus_phage_S_CRM01, Cronobacter_phage_S13, Bacillus_phage_vB_BpuM_BpSp, and Yersinia_phage_phiR1_37 were differed significantly among the three weaning periods.

### Alterations in microbial function among three weaning periods

**Fig. 3:**
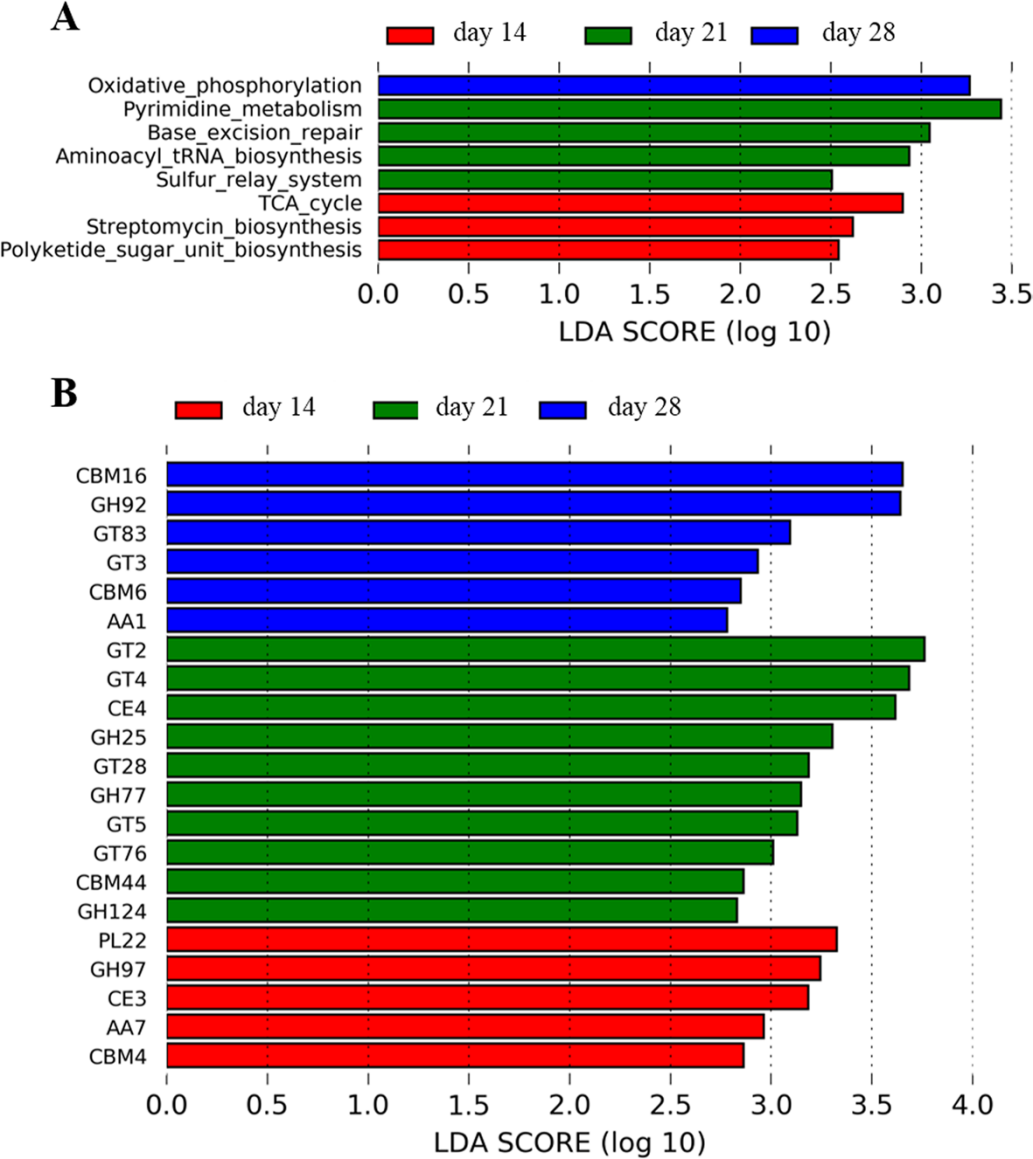
The significantly different KEGG pathways and CAZymes. **A** Significantly different KEGG pathways. **B** Significantly different CAZymes

The functional capacity of the gut microbiome in relation to porcine weaning periods was investigated using metagenomic sequencing data. We classified the microbial gene catalog from our analysis by aligning them to the KEGG database and Carbohydrate-Active enZYmes database (CAZy). We identified a total of eight KEGG functional terms that were distinctly enriched among the three weaning periods (Fig. 3A and Supplementary Table S2). Oxidative phosphorylation was the only more abundant functional terms in the day 28 group, while among seven other pathways, the citrate cycle (TCA cycle), streptomycin biosynthesis, and polyketide sugar unit biosynthesis, and pyrimidine metabolism, base excision repair, aminoacyl-tRNA biosynthesis and sulfur relay system were more enriched in the day 14 and day 21 groups, respectively. For CAZymes, a total of 21 CAZymes were identified to have significantly different abundances among the three weaning periods (Fig. 3B and Supplementary Table S3), including five and ten CAZymes involved in the metabolism of xylan, galactose and starch enriched in the day 14 and day 21 groups, respectively. And the six CAZymes with significantly higher abundance in the gut microbiome of day 28 group were demonstrated to be mainly involved in the binding to cellulose and glucomannan (CBM16).

**Fig. 4:**
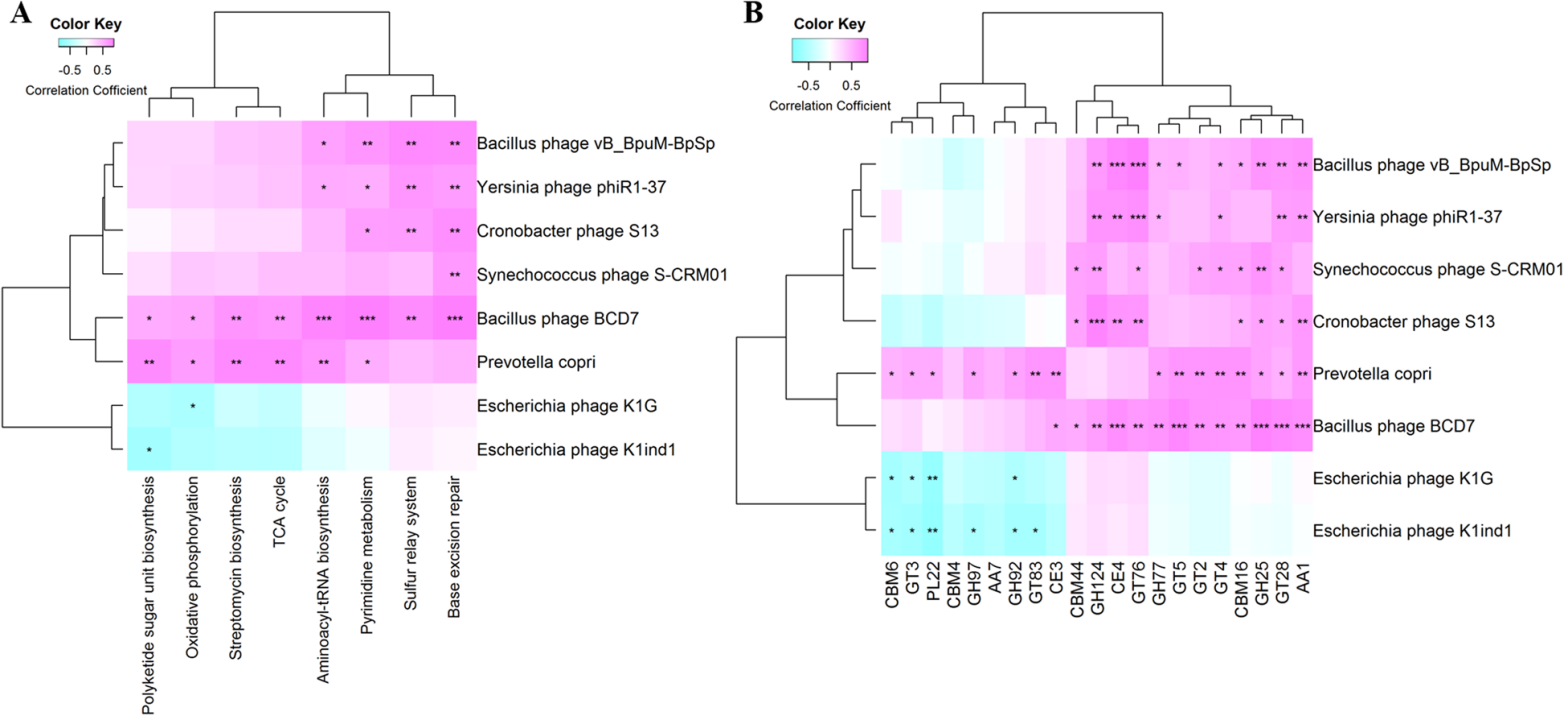
The relationship between the differential species and differential KEGG pathways or differential CAZymes. **A** The relationship between the differential species and differential KEGG pathways. **B** The relationship between the differential species and differential CAZymes. The differential species included one and seven overlap bacterial and bacteriophages through LEfSe and random forest analysis, respectively. The *X*-axis represents the differential species. The *Y*-axis indicates the differential KEGG pathways or CAZymes. * *P* < 0.05, ** *P* < 0.01, and *** *P* < 0.001

Furthermore, to determine the relationship between the differential species (one and seven overlap bacterial and bacteriophages through LEfSe and random forest analysis, respectively) and differential KEGG pathways, as well as between the differential species and differential CAZymes, a Spearman correlation analysis was used. The results indicated that *P. copri* was positively and significantly correlated with most of the differential KEGG pathways (except the base excision repair pathway and the sulfur relay system) (Fig. 4A). We also found that all of the differential KEGG pathways were positively and significantly correlated with the Bacillus_phage_BCD7 (Fig. 4A). With regard to CAZymes enrichment, we found that most of CAZymes were positively and significantly correlated with *P. copri* (Fig. 4B). The correlation analysis also indicated the contribution of the bacteria species to the changes of CAZymes.

### Differential serum metabolite profiles among the three weaning periods

**Fig. 5:**
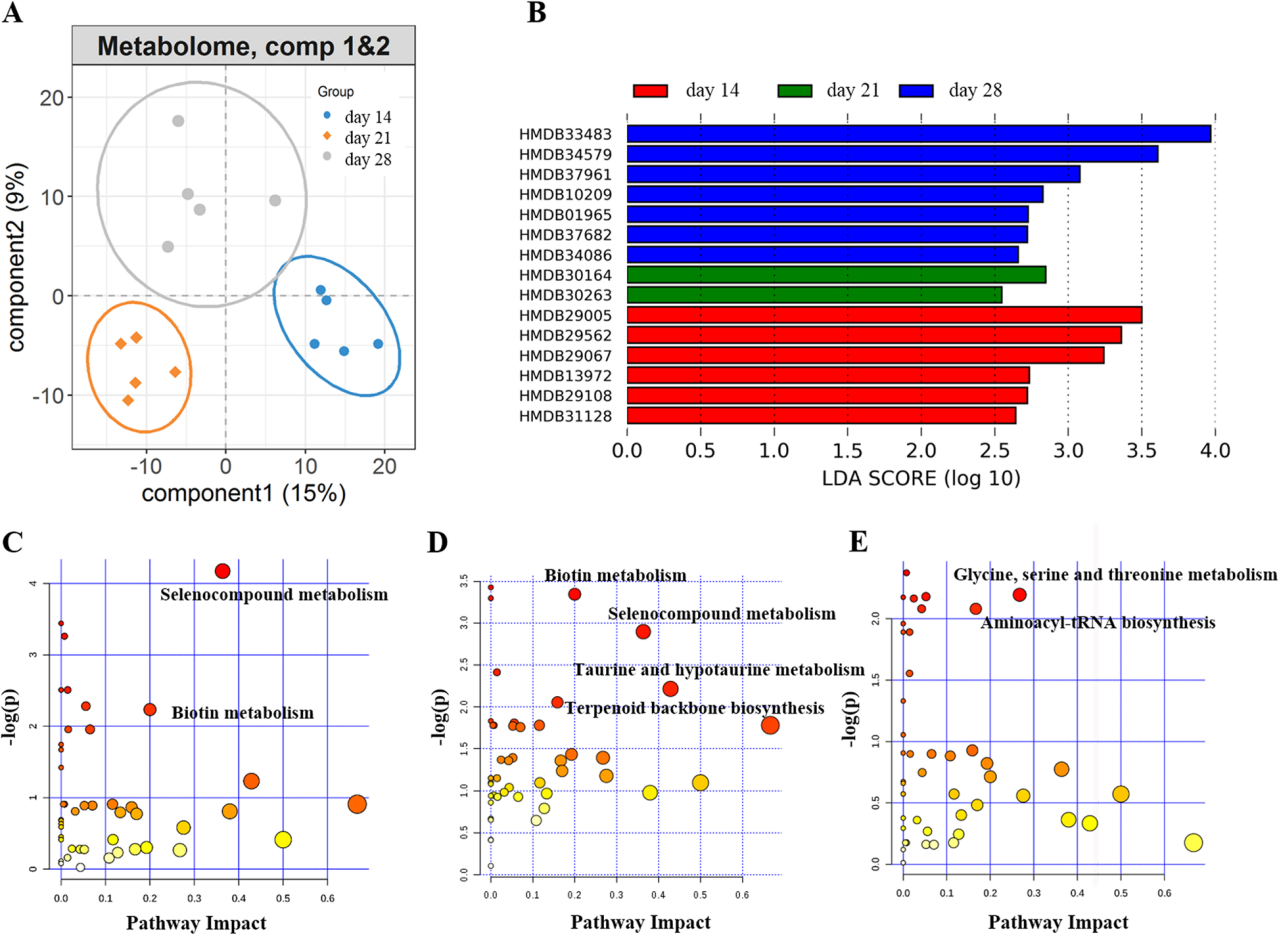
Changes in the serum metabolome and functional enrichment of serum metabolite features. **A** PLS-DA plot of serum metabolite profiles, which indicates the significant differentiation of serum metabolite profiles among the three weaning periods. **B** The differential serum metabolite features. KEGG pathway enrichment analysis based on the metabolite features altered among the three weaning periods (**C-E**). The X-axis and the size of the dots indicate the pathway impact of various altered metabolite features (the sum of the importance measures of the matched metabolites normalized to the sum of the importance measures of all metabolites in each pathway), and the Y-axis shows the *P*-value obtained for each pathway in the enrichment analysis. The size and color of the dots indicate the overall pathway impact

To systematically evaluate shifts in the host serum metabolome among the three weaning periods, the pig serum metabolomic profiles were determined using UHPLC-MS/MS. After normalization, we obtained a total of 831 metabolites. And an obvious shift in the global metabolome was observed among the three weaning periods (Fig. 5A). Specifically, we identified a total of 15 metabolite features showing distinct enrichment patterns among the three weaning periods (Fig. 5B and Supplementary Table S4). These enriched metabolites included six metabolite features that were significantly enriched in the day 14 group, two metabolite features that were significantly enriched in the day 21 group, and seven metabolite features that were significantly enriched in the day 28 group. For example, phenylalanyl-threonine (HMDB29005) and N-gamma-L-Glutamyl-L-phenylalanine (HMDB29562), cyclonormammein (HMDB30164) and rhazidigenine Nb-oxide (HMDB30263), 6,7-Dihydro-4-(hydroxymethyl)-2-(p-hydroxyphenethyl)-7-methyl-5H-2-pyrindinium (HMDB33483) and 8-O-Methyloblongine (HMDB34579) were enriched in the day 14, day 21 and day 28 groups, respectively. Furthermore, all metabolite features were analyzed using KEGG pathway enrichment analysis. Compared with the day 14 group and day 21 group, the metabolites that were significantly enriched were in the KEGG pathways for selenocompound metabolism and biotin metabolism (Fig. 5C). When comparing with day 14 group and day 28 group, the metabolites that were significantly enriched were in the KEGG pathways for biotin, selenocompound, taurine and hypotaurine metabolism, and terpenoid backbone biosynthesis (Fig. 5D). Furthermore, we found there were metabolites that were significantly enriched in the KEGG pathways for glycine, serine and threonine metabolism and aminoacyl-tRNA biosynthesis when comparing between the day 21 and day 28 groups (Fig. 5E).

### Co-occurrence analysis among the gut bacteria, bacteriophages and serum metabolites

**Fig. 6:**
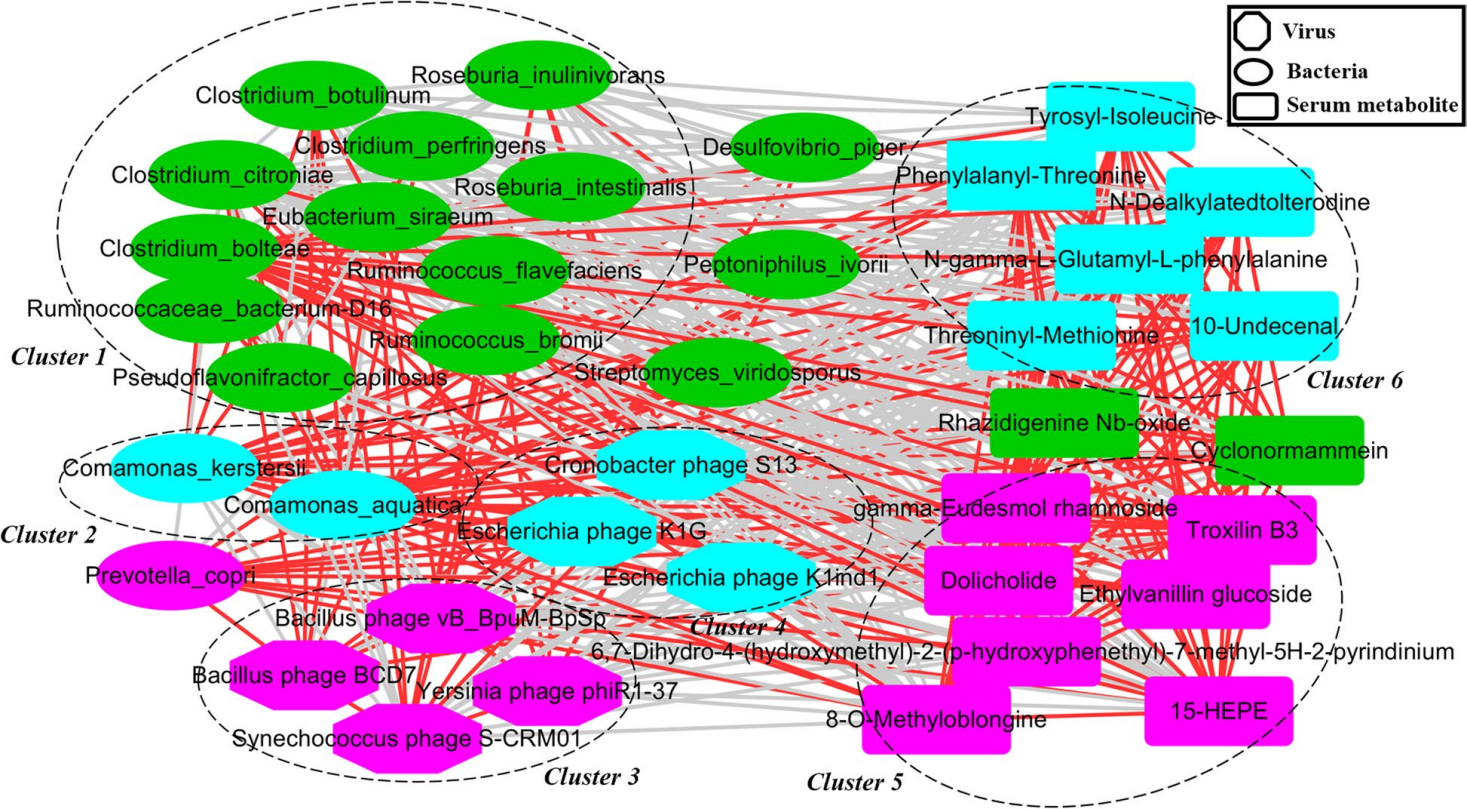
A co-occurrence network constructed with gut bacterial species, bacteriophages and serum metabolites showing different abundances among the three weaning periods. Green, blue and purple dots indicate the day 14 group, day 21 group and day 28 group, respectively. Edges between nodes indicate Spearman’s negative (light gray) or positive (light red) correlation

We further explored the potential correlations among differential bacterial species, bacteriophages and serum metabolites by co-occurrence network analysis. Bacterial species were found to form strong and broad co-occurring relationships with serum metabolites; bacteriophages, on the other hand, displayed only mild correlations with bacterial species and serum metabolites (Fig. 6). The differential bacterial species, bacteriophages and serum metabolites were mainly aggregated into six clusters in this network. The bacterial species belonging to cluster 1 was enriched in day 14 group. And the bacterial species enriched in day 21 group were included in the cluster 2. Interesting, the *P. copri* was positively and significantly correlated with only one bacteriophages (Bacillus_phage_BCD7) and most of serum metabolites enriched in day 28 group.

## Discussion

Gut microbiota has been treated as an important organ of body. It is influenced by diverse factors that include different regions, environments, dietary styles and genetic backgrounds [25–27]. Several studies have been focused on how the gut microbiome develops and changes during the suckling and weaning periods [22–24, 28]. However, there were small in scale and used 16S rRNA gene sequencing and/or metagenomic sequencing and/or no metabolomic profiles. In this study, we compared the bacterial and virus composition of the gut microbiome by metagenomic sequencing, and identified bacterial and virus species associated with three weaning periods in piglets. We found that the changes of gut bacteria and bacteriophages were correlated with the shifts of serum metabolome, and then, should influence the piglets suckling and weaning.

*P. copri* was the most significantly different species among three weaning periods. Random forest analysis also identified *P. copri* as a weaning-biased bacterium at the species level. Many studies have suggested that *P. copri* could induce insulin resistance, aggravate glucose intolerance, and alter complex carbohydrate degradation [29, 30]. Nguyen et al. showed that piglets with access to soil during lactation were switched to a plant-based diet after weaning, and had gut microbiomes enriched for *Prevotella* and maturated more quickly [31]. The bacterial species associated with the day 21 group were found to belong to *Roseburia* and *Ruminococcaceae*, which are capable of producing short-chain fatty acids (SCFA) by fermentation of dietary polysaccharides [32, 33]. These microbes, including *Prevotella*, are efficient at degrading dietary fibers and producing SCFA, indicating a shift toward a more adult pig-like intestinal environment associated with increased functional capability for carbohydrate degradation.

Through LEfSe and random forest analysis, seven differential bacteriophages were found overlap. Expressly Bacillus_phage_BCD7, the only differential bacteriophages, was significantly and positively correlated with *P. copri* enriched in day 28 group. The result indicated that bacteria-phage interactions might importantly for mitigating piglet adaptation during the weaning transition. Rodriguez-Valera et al*.* and Enault et al*.* also found that phages can increase bacterial growth and fitness by providing bacteria with genes for touching upon polysaccharide, toxin, carbohydrate metabolism and antibiotic resistance [18, 19]. In addition, we also found that some differential bacteriophages were correlated with some differential serum metabolites, suggesting that these bacteriophages may indirectly affect serum metabolites. However, it should be further confirmed and verified in future studies.

Functional capacity analysis revealed that the gut microbiome of piglets after weaning might be more capable of utilizing dietary protein than that of piglets prior to weaning. Interesting, we also observed that KEGG pathways and CAZymes related to energy metabolism were significantly positively associated with *P. copri*. Oxidative phosphorylation is the major ATP producing pathway in humans [34]. It is also well-established that carbohydrate degradation is closely related to oxidative phosphorylation. Cells growing in the presence of glucose utilize both glycolysis and oxidative phosphorylation for ATP production, whereas cells growing in galactose are almost entirely dependent on oxidative phosphorylation [35, 36]. Interestingly, the differential metabolites were enriched in pathways related to the functional capacity of the gut microbiome, such as aminoacyl-tRNA biosynthesis, suggesting a relationship between shifts in the gut microbiome and the host serum metabolome. The function of aminoacyl-tRNA synthesis is to precisely match amino acids with tRNAs containing the corresponding anticodon [37]. Others like glycine, serine and threonine metabolism might relate to utilizing the glucose [38].

## Conclusions

In this study, we found that fecal bacterial and virus composition, functional capacity, and serum metabolites were significantly changed among three weaning periods in piglets. The *P. copri* is a key bacterial species for mitigating piglet adaptation during the weaning transition. It was significantly correlated with the changes of KEGG pathways and CAZymes. Enriched metabolites were found in pathways that are matched to the functional capacity of the gut microbiome (e.g. aminoacyl-tRNA biosynthesis). All results suggest that gut microbes, such as *P. copri* may affect piglet weaning transition through changing the serum metabolites. The results from this study indicate that host-microbial interactions during the weaning transition impact host metabolism, leading to beneficial host changes among three weaning periods. However, this study has several limitations, the number of repetitions was five (two male and three female) and these results were only established based on association studies, and the causality and underlying mechanisms have not been elucidated. These questions will need to be addressed in future studies.

## Materials and methods

### Experimental animals and sample collection

Five Large White piglets (two male and three female) across three age strata were studied. All piglets stayed with their mothers during the suckling period and allowed to nurse freely until weaning after 21 days of age. Then all piglets were provided the same commercial formula diet and clean water ad libitum. According to previous studies [24, 39], we chose seven days before or after weaning day for other two periods. Fresh feces from all piglets were collected from each animal’s anus by rectal massage at 14 days of age (day 14 group), 21 days (the day of weaning, day 21 group), and 28 days of age (day 28 group). All piglets were no obvious disease or diarrhea and received no probiotic or antibiotic therapy during the period from birth to the end of this study. Fecal samples were immediately snap-frozen in liquid nitrogen for transportation and then stored at − 80 °C for later use. Blood samples were also collected from each experimental pig at the time of sampling.

### Microbial DNA extraction

Microbial DNA was extracted from fecal samples using the QIAamp Fast DNA Stool Mini Kit (Qiagen, Germany) according to the manufacturer’s instructions. The concentration of DNA was determined using a Nanodrop-2000 spectrophotometer (Thermo Fisher Scientific, MA, US) and the DNA purity was confirmed by 0.8% (w/v) agarose gel electrophoresis. All DNA samples were stored at -20 °C until further processing.

### Metagenomic sequencing analysis

Metagenomic sequencing of the 15 fecal microbial DNA samples was performed using a Novaseq-PE150 platform. Sequencing libraries were generated using NEB Next® Ultra™ DNA Library Prep Kit (NEB, USA) following manufacturer’s recommendations with an insert size of 350 base pairs (bp), and index codes were added to attribute sequences of each sample. The libraries were analyzed for size distribution using an Agilent 2100 Bioanalyzer, quantified using real-time PCR, and sequenced on a Novaseq 6000 platform (Illumina, USA) with pair-end 150 bp strategy.

The raw sequencing data was preprocessed using Readfq to acquire clean data for subsequent analysis. The clean sequence reads were further blasted against the pig reference genome (Sscrofa 11.1) using Bowtie2.2.4 software [40] to filter out the host pollution. Then, the clean data was assembled using SOAPdenovo software (v.2.21) [41, 42]. The contigs with more than 500 bp in length were used to predict open reading frames (ORF) using MetaGeneMark (V2.10) [31, 41]. For ORF predicted, CD-HIT [43] software is adopted to redundancy and obtain the unique initial gene catalogue and using Bowtie2.2.4 to filter for the genes for which the number of reads was less than two in each sample and obtained the gene catalogue (Unigenes) eventually used for all subsequent analysis. DIAMOND [29] software was used to blast the Unigenes to the sequences of bacteria and viruses, all of which were extracted from the NCBI NR database. We chose the result where the e value ≤ the smallest e value × 10 [30] to take the LCA algorithm which is applied to system classification of MEGAN [32] software to make sure the species annotation information of sequences. Functional annotations were performed by aligning the putative amino acid sequences which were translated from the predicted genes exclude KEGG [34] and CAZy [35] database.

### Determination of the metabolomic profiles of porcine serum samples

Fifteen serum samples were used for untargeted metabolomic analysis. The method for performing untargeted metabolomics was performed as described by Want et al. [44]. Briefly, porcine serum samples (100 μL) and prechilled methanol (400 μL) were mixed by well vortexing. Then, LC–MS/MS analyses were performed using a Vanquish UHPLC system (Thermo Fisher) coupled with an Orbitrap Q Exactive series mass spectrometer (Thermo Fisher). The raw data files generated by UHPLC-MS/MS were processed using Compound Discoverer 3.0 (CD3.0, Thermo Fisher) to perform peak alignment, peak picking, and quantitation for each metabolite. Statistical analysis of the results was performed using R (R version R-3.4.3), Python (Python 2.7.6 version), and CentOS (CentOS release 6.6). When data were not normally distributed, normal transformations were attempted using an area normalization method.

## Statistical analysis

Sparse Correlations for Compositional data (SparCC) was employed to determine co-abundance (positive) and co-exclusion (negative) relationships among differential bacterial species, bacteriophages and serum metabolites based on their relative abundances [45]. Network analysis was performed and visualized using Cytoscape (version 3.6.1). To identify the different abundances among groups, linear discriminant analysis (LDA) and effect size (LEfSe) analysis were performed under the condition *α* = 0.01, with an LDA score of at least 2.50 [45]. Story’s FDR was used to correct the multiple tests. Partial Least Squares-Discriminant analysis (PLS-DA) was performed to evaluate gut bacterial species, gut bacteriophages, and serum metabolite profiles among three weaning periods [46].

## Supplementary Information

**Additional file 1:** **Table S1.** The sequence assembly analysis for shotgunmetagenomic sequencing.** Table S2.** The differential KEGG pathways amongthree weaning periods. **Table S3.** The differential CAZy family amongthree weaning periods. **Table S4.** The differential metabolites amongthree weaning periods.

## Data Availability

All of the data generated or analyzed during this study are available from the corresponding author on reasonable request.

## Abbreviations

NT: Nucleotide
CAZy: Carbohydrate-Active enZYmes database
SCFA: Short-chain fatty acids
SparCC: Sparse Correlations for Compositional
LDA: Linear discriminant analysis
LEfSe: Linear discriminant analysis effect size
PLS-DA: Partial Least Squares-Discriminant analysis
