# Supplementary material for: Identifying biomarkers of the gut bacteria, bacteriophages and serum metabolites associated with three weaning periods in piglets
Source: BMC Vet Res. 2022 Mar 17;18:104. doi: 10.1186/s12917-022-03203-w (PMC8928592; doi:10.1186/s12917-022-03203-w)
Supplement: Supplementary file 1 — Additional file 1: Table S1. The sequence assembly analysis for shotgunmetagenomic sequencing. Table S2. The differential KEGG pathways amongthree weaning periods. Table S3. The differential CAZy family amongthree weaning periods. Table S4. The differential metabolites amongthree weaning periods. [file 12917_2022_3203_MOESM1_ESM.docx]

**Table S1. The sequence assembly analysis for shotgun metagenomic sequencing**

| **Sample ID** | **Group** | **Total len.(bp)** | **Num.** | **Average len.(bp)** | **N50 Len.(bp)** | **N90 Len.(bp)** | **Max len.(bp)** |
| --- | --- | --- | --- | --- | --- | --- | --- |
| sample01 | day 14 | 154,430,111 | 79,172 | 1,950.56 | 3,690 | 685 | 465,557 |
| sample02 | day 14 | 193,422,424 | 87,987 | 2,198.31 | 4,782 | 733 | 768,060 |
| sample03 | day 14 | 172,716,624 | 69,192 | 2,496.19 | 8,609 | 760 | 393,425 |
| sample04 | day 14 | 197,818,435 | 101,366 | 1,951.53 | 3,483 | 698 | 457,626 |
| sample05 | day 14 | 106,318,122 | 48,047 | 2,212.79 | 5,458 | 722 | 380,442 |
| sample06 | day 21 | 264,156,618 | 137,189 | 1,925.49 | 3,370 | 689 | 311,606 |
| sample07 | day 21 | 260,171,306 | 133,047 | 1,955.48 | 3,746 | 686 | 340,330 |
| sample08 | day 21 | 195,592,579 | 83,442 | 2,344.05 | 5,024 | 774 | 254,666 |
| sample09 | day 21 | 197,707,695 | 89,314 | 2,213.62 | 5,439 | 713 | 592,496 |
| sample10 | day 21 | 184,559,247 | 99,310 | 1,858.42 | 3,321 | 663 | 452,237 |
| sample11 | day 28 | 291,761,609 | 148,095 | 1,970.10 | 4,142 | 682 | 286,734 |
| sample12 | day 28 | 253,358,141 | 117,510 | 2,156.06 | 4,826 | 713 | 761,807 |
| sample13 | day 28 | 263,634,987 | 132,116 | 1,995.48 | 3,993 | 694 | 722,641 |
| sample14 | day 28 | 256,646,172 | 124,006 | 2,069.63 | 4,712 | 693 | 382,918 |
| sample15 | day 28 | 239,910,236 | 116,367 | 2,061.67 | 4,390 | 705 | 308,611 |

**Table S2. The differential KEGG pathways among three weaning periods**

| **Function terms** | **Annotation** | **Group** | **LDA value** | **P value** |
| --- | --- | --- | --- | --- |
| ko00020 | Citrate cycle (TCA cycle) | day 14 | 2.90 | 0.0242 |
| ko00521 | Streptomycin biosynthesis | day 14 | 2.62 | 0.0226 |
| ko00523 | Polyketide sugar unit biosynthesis | day 14 | 2.54 | 0.0255 |
| ko00240 | Pyrimidine metabolism | day 21 | 3.44 | 0.0347 |
| ko03410 | Base excision repair | day 21 | 3.05 | 0.0108 |
| ko00970 | Aminoacyl-tRNA biosynthesis | day 21 | 2.93 | 0.0324 |
| ko04122 | Sulfur relay system | day 21 | 2.50 | 0.0265 |
| ko00190 | Oxidative phosphorylation | day 28 | 3.27 | 0.0324 |

**Table S3. The differential CAZy family among three weaning periods**

| **CAZy family** | **Group** | **LDA value** | **P value** |
| --- | --- | --- | --- |
| PL22 | day 14 | 3.33 | 0.031 |
| GH97 | day 14 | 3.24 | 0.027 |
| CE3 | day 14 | 3.18 | 0.031 |
| AA7 | day 14 | 2.96 | 0.034 |
| CBM4 | day 14 | 2.86 | 0.026 |
| GT2 | day 21 | 3.76 | 0.027 |
| GT4 | day 21 | 3.68 | 0.032 |
| CE4 | day 21 | 3.62 | 0.012 |
| GH25 | day 21 | 3.30 | 0.015 |
| GT28 | day 21 | 3.19 | 0.027 |
| GH77 | day 21 | 3.15 | 0.026 |
| GT5 | day 21 | 3.13 | 0.031 |
| GT76 | day 21 | 3.01 | 0.027 |
| CBM44 | day 21 | 2.86 | 0.035 |
| GH124 | day 21 | 2.83 | 0.026 |
| CBM16 | day 28 | 3.65 | 0.027 |
| GH92 | day 28 | 3.64 | 0.019 |
| GT83 | day 28 | 3.09 | 0.013 |
| GT3 | day 28 | 2.93 | 0.009 |
| CBM6 | day 28 | 2.85 | 0.013 |
| AA1 | day 28 | 2.78 | 0.013 |

**Table S4. The differential metabolites among three weaning periods**

| **Retention time (RT)–m/z** | **HMDB** | **Putative compound** | **Group** | **LDA value** | **P value** |
| --- | --- | --- | --- | --- | --- |
| 13.28_265.1479 m/z | HMDB33483 | 6,7-Dihydro-4-(hydroxymethyl)-2-(p-hydroxyphenethyl)-7-methyl-5H-2-pyrindinium | day 28 | 3.97 | 0.018 |
| 13.07_309.1741 m/z | HMDB34579 | 8-O-Methyloblongine | day 28 | 3.61 | 0.008 |
| 3.41_266.1274 n | HMDB29005 | Phenylalanyl-Threonine | day 14 | 3.50 | 0.018 |
| 4.29_294.1242 n | HMDB29562 | N-gamma-L-Glutamyl-L-phenylalanine | day 14 | 3.36 | 0.019 |
| 3.94_250.0966 n | HMDB29067 | Threoninyl-Methionine | day 14 | 3.24 | 0.010 |
| 13.28_349.2414 m/z | HMDB37961 | gamma-Eudesmol rhamnoside | day 28 | 3.08 | 0.024 |
| 12.33_299.2016 m/z | HMDB10209 | 15-HEPE | day 28 | 2.83 | 0.009 |
| 10.41_355.1578 m/z | HMDB30164 | Cyclonormammein | day 21 | 2.79 | 0.015 |
| 2.72_284.1979 m/z | HMDB13972 | N-Dealkylatedtolterodine | day 14 | 2.74 | 0.006 |
| 13.28_335.2256 m/z | HMDB01965 | Troxilin B3 | day 28 | 2.73 | 0.009 |
| 4.58_309.1013 m/z | HMDB37682 | Ethylvanillin glucoside | day 28 | 2.72 | 0.025 |
| 3.41_312.1934 m/z | HMDB29108 | Tyrosyl-Isoleucine | day 14 | 2.72 | 0.012 |
| 13.21_477.3247 m/z | HMDB34086 | Dolicholide | day 28 | 2.66 | 0.024 |
| 15.83_169.1594 m/z | HMDB31128 | 10-Undecenal | day 14 | 2.64 | 0.032 |
| 10.41_313.1907m/z | HMDB30263 | Rhazidigenine Nb-oxide | day 21 | 2.55 | 0.018 |
